# The effect of four different freezing conditions and time in frozen storage on the concentration of commonly measured growth factors and enzymes in equine platelet-rich plasma over six months

**DOI:** 10.1186/s12917-019-2040-4

**Published:** 2019-08-14

**Authors:** Andrew K. McClain, Taralyn M. McCarrel

**Affiliations:** Department of Large Animal Clinical Sciences, University of Florida College of Veterinary Medicine, 2015 SW 16th Avenue, Gainesville, FL 32610 USA

**Keywords:** Horse, Platelet rich-plasma, Storage, Freezing, Growth factor

## Abstract

**Background:**

Platelet-rich plasma (PRP) is a therapeutic biologic that is used for treatment of musculoskeletal pathologies in equine athletes. Due to the expense of PRP kits, and the volumes obtained, freezing aliquots for future dosing is common. Aliquots of PRP are also commonly frozen for later analysis of growth factor concentrations in in vitro research. A variety of freezing methods are used and storage duration until analysis is often not reported. The optimal frozen storage conditions and duration to maintain concentrations of commonly measured growth factors and enzymes in PRP are unknown. Our objectives were two-fold. First, to determine the effect of a single freeze-thaw cycle on PRP protein concentrations and establish their baseline levels. Second, to evaluate the effect of storage in -20 °C automatic defrost freezer, − 20 °C manual defrost freezer, − 80 °C manual defrost freezer, and liquid nitrogen for 1, 3, and 6 months on PRP protein concentrations, compared to the established baseline concentrations.

**Results:**

Fold-change between fresh activated and snap frozen PRP were analyzed using paired t-test. A snap frozen-thaw cycle resulted in increased MMP-9 (*p* = 0.0021), and a small significant decrease in TGF-β1 (*p* = 0.0162), while IGF-1 and PDGF-BB were unchanged compared to fresh activated PRP. Fold-change over time within storage method were analyzed using repeated measures ANOVA and Tukey post-hoc test. IGF-1 decreased in all conditions (*p* < 0.0001). At all time-points at -20 °C (*p* < 0.0001), and at 3 and 6 months at -80 °C (*p* < 0.0070), PDGF-BB decreased. TGF- β1 was unchanged or increased after 6 months (*p* < 0.0085). MMP-9 decreased at 3-months at -20 °C, and at all times at -80 °C and in liquid nitrogen compared to snap frozen (*p* < 0.0001).

**Conclusions:**

The protein profile of equine frozen-stored PRP differs from fresh PRP. For clinical applications equine PRP can be stored at -80 °C for 1 month or in liquid nitrogen for 6 months to maintain PDGF-BB and TGF-β1 concentration, but IGF-1 concentrations will be reduced. The storage temperature and duration should be reported in studies measuring protein concentrations in PRP. To accurately measure IGF-1 concentrations, PRP samples should be analyzed immediately.

**Electronic supplementary material:**

The online version of this article (10.1186/s12917-019-2040-4) contains supplementary material, which is available to authorized users.

## Background

Platelet-rich plasma (PRP) is a regenerative therapy prepared from blood that contains increased concentrations of platelets compared to whole blood. In the horse, PRP has been used for over a decade for treatment of musculoskeletal injury and disease [1–3]. While much focus is on the platelets in PRP, it is also comprised of plasma, white blood cells (WBC), and red blood cells (RBC). Ultimately, it is the proteins contained within the respective constituents that exert PRPs biologic effects [4–6]. Growth factors such as insulin-like growth factor (IGF), platelet-derived growth factor (PDGF), and transforming growth factor (TGF), are known to have chemotactic, mitogenic, and matrix synthesis effects in tissues. Inflammatory mediators and catabolic enzymes such as matrix metalloproteinase-9 (MMP-9) are also present in PRP and correlate with WBC concentration [7].

Validating PRP for various indications has been challenging due to wide variability in preparation and delivery. While many questions remain, studies in horses have evaluated the composition of PRP generated by a variety of systems [8], the effect of platelet [9] and WBC [10, 11] concentration on tendon explants, and the effect of various activators on growth factor release [12–14]. Many of the beneficial growth factors in PRP are sequestered in platelet alpha granules, and platelet activation or lysis is required for their release. While freezing and thawing PRP has been used as a method for growth factor release in several studies [11, 15], PRP may also be frozen for later clinical use [1]. Preparation of PRP using manufactured kits is expensive and often yields more PRP than is needed for a single treatment. In addition, there is recent increasing interest in autologous or pooled allogenic platelet lysates to replace fetal bovine serum (FBS) in cell culture for clinical treatment of horses to eliminate the immunogenic effects of FBS [16]. For these reasons the practice of freezing PRP is common in both veterinary and human sports medicine [17].

While there are anecdotal references to frozen storage of PRP, and growth factor concentrations in frozen-thawed PRP have been measured, there have been no studies comparing growth factor concentrations in fresh PRP and frozen PRP stored for an extended period of time. Furthermore, throughout the literature it is frequently reported that PRP samples are collected and stored in various temperatures, such as − 20 °C [12, 13], − 30 °C [18] -80 °C [6, 11, 13, 19], and − 82 °C [14]. Further, the duration of frozen storage from the time of collection until analysis is not typically reported. The consequences of such storage practices without validation for the substances being measured is that the results reported may be inaccurate due to artifactual changes during storage.

The first objective of this study was to determine the effect of a single snap freeze-thaw cycle and activation (LN0) on TGF-β1, PDGF-BB, IGF-1, and MMP-9 concentrations in equine PRP compared to fresh activated PRP (PRP0). The second objective was to evaluate the effect of storage for 1, 3, and 6 months within 4 different freezing conditions on the same proteins as in objective 1. The storage methods included in the study were a -20 °C automatic defrost freezer (− 20A), a -20 °C manual defrost freezer (− 20 M), a -80 °C manual defrost freezer (− 80 M), and liquid nitrogen (LN). We hypothesized that the concentration of proteins measured in LN0 would not differ from PRP0. Further, we hypothesize that protein concentrations in PRP stored in -20A would decrease with time while there would be no change in protein concentrations in PRP stored under any other condition for 6 months when compared to PRP0.

## Results

### Hematology

Platelet and WBC concentrations were significantly increased and hematocrit significantly decreased in PRP compared to whole blood, confirming the production of PRP (Table 1).

**Table 1:** Platelet and white blood cell (WBC) concentrations, and hematocrit in blood and platelet-rich plasma (PRP)

	WBC (× 10^3^/μl)	Platelet (× 10^3^/μl)	Hematocrit (%)
Whole Blood	5.3 ± 0.3	107.7 ± 2.7	31.4 ± 3.1
PRP	11.7 ± 1.8 †	534.8 ± 19.8 †	2.8 ± 1.8 †
*p*-value	0.0100	< 0.0001	< 0.0001

Values reported as mean (*n* = 6) ± standard error. (†) indicates significant difference between PRP and whole blood using a paired t-test, *p* < 0.05

### Freezer temperature

Throughout the 6-month study, the RC-5 temperature data logger measured a maximum temperature of − 3 °C, a minimum of − 25.3 °C, and an average of − 20.4 °C in -20A. The temperature consistently increased to just below freezing every 16 h. Time to defrost from average temperature to minimum temperature ranged from 30 to 45 min, and time to return to average temperature ranged from 45 to 60 min. All other freezers maintained their respective temperatures and were monitored continuously on the institutional alarm system.

### Effect of snap freeze-thaw on protein concentrations

Quantitation of each protein of interest in PRP0 and LN0 was performed to determine the effect of a single snap freeze-thaw cycle, without the element of time, on protein concentrations (Table 2). There was no change in IGF-1 and PDGF-BB concentrations between PRP0 and LN0. Thus only PRP0 was used to compare 1-, 3-, and 6-month data to. Concentrations of TGF-β1 significantly decreased in LN0 compared to PRP0. However, the decrease in TGF- β1 was small and unlikely to be clinically significant; therefore, PRP0 was used to compare 1-, 3-, and 6-month data to. Concentrations of MMP-9 significantly increased in LN0 compared to PRP0. As a result, for MMP-9 only, PRP0 and LN0 were included in statistical analysis to compare 1-, 3- and 6- month data.

**Table 2:** Protein concentrations in fresh activated platelet-rich plasma (PRP0) and liquid nitrogen frozen-thawed activated PRP (LN0)

	PRP0	LN0	LN0/PRP0	*p*-value
IGF-1	340 ± 21.5 ng/ml	333 ± 24.3 ng/ml	0.98	0.3141
PDGF-BB	6058 ± 696 pg/ml	4942 ± 397 pg/ml	0.85	0.1211
TGF-β1	4671 ± 896 pg/ml	4329 ± 911 pg/ml	0.92 †	0.0162
MMP-9	11,156 ± 2743 pg/ml	21,762 ± 3457 pg/ml	2.19 †	0.0021

Values reported as mean (*n* = 6) ± standard error. (†) indicates significant difference between PRP0 and LN0 using a paired t-test, *p* < 0.05. Insulin-like growth factor (IGF-1), platelet-derived growth factor-BB (PDGF-BB), transforming growth factor-β1 (TGF-β1), matrix metalloproteinase-9 (MMP-9), fold-change of liquid nitrogen frozen-thawed activated PRP at time 0 to fresh activated PRP at time 0 (LN0/PRP0)

### Protein concentrations over time

**Fig. 1:**
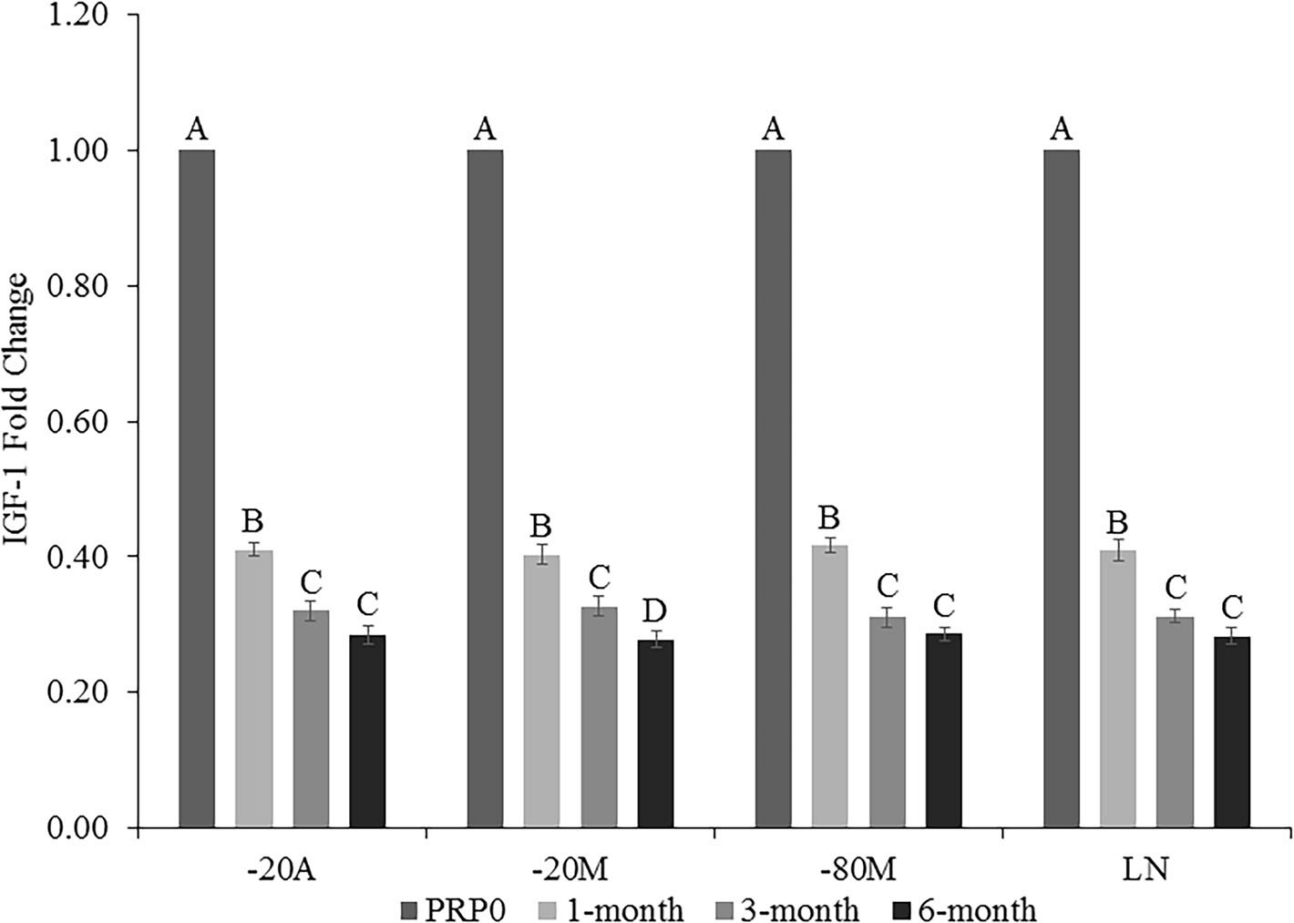
Insulin-like growth factor (IGF-1) fold-change in platelet-rich plasma (PRP) at 1, 3, and 6 months of storage in − 20 °C automatic defrost freezer (− 20A), − 20 °C manual defrost freezer (− 20 M), − 80 °C manual defrost freezer (− 80 M), and liquid nitrogen (LN) compared to baseline fresh activated PRP at time 0 (PRP0). All samples were activated with bovine thrombin and calcium chloride prior to growth factor quantification at each time point. Bars represent mean (*n* = 6) ± standard error. Differing letters indicate a significant difference between groups within storage method, using a repeated measures ANOVA with a Tukey post-hoc test, *p* < 0.05

There was a significant decrease in IGF-1 concentration in all storage conditions (*p < 0.0001*) at all time points compared to PRP0 (Fig. 1). There was an additional further decrease in IGF-1 concentration at 3 months in all storage conditions, and an additional decrease at 6 months in -20 M.

**Fig. 2:**
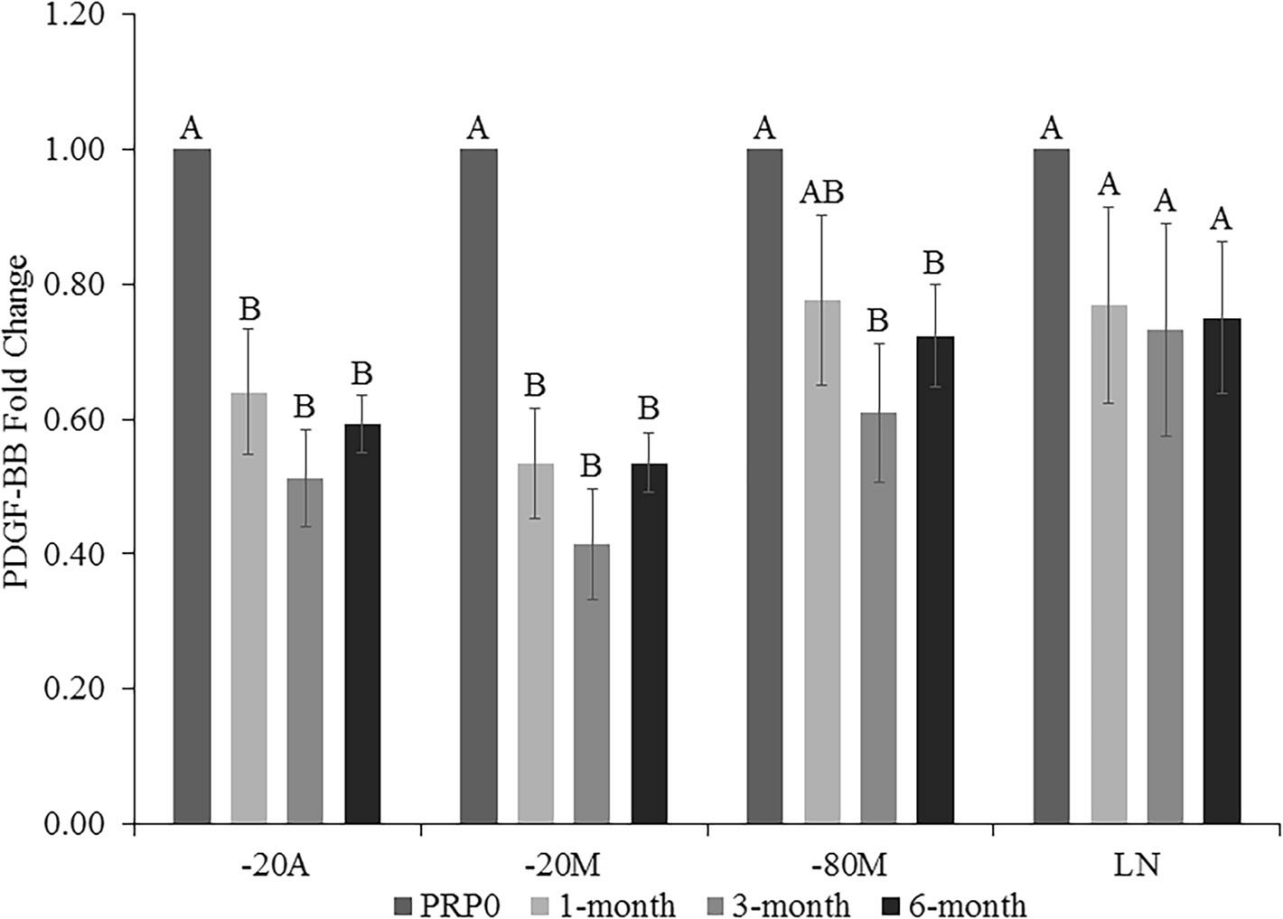
Platelet derived growth factor-BB (PDGF-BB) fold change in platelet-rich plasma (PRP) at 1, 3, and 6 months of storage in − 20 °C automatic defrost freezer (− 20A), − 20 °C manual defrost freezer (− 20 M), − 80 °C manual defrost freezer (− 80 M), and liquid nitrogen (LN) compared to baseline fresh activated PRP at time 0 (PRP0). All samples were activated with bovine thrombin and calcium chloride prior to growth factor quantification at each time point. Bars represent mean (*n* = 6) ± standard error. Differing letters indicate a significant difference between groups within storage method, using a repeated measures ANOVA with a Tukey post-hoc test, *p* < 0.05

There was a significant decrease in PDGF-BB after 1 month in storage in both -20A (*p < 0.0001*) and -20 M (*p < 0.0001*) compared to PRP0 with no further decrease thereafter (Fig. 2). The concentration of PDGF-BB was significantly decreased at 3- and 6-month time points compared to PRP0 in aliquots stored in − 80 M (*p < 0.0070*). There was no difference in PDGF-BB concentration at any time in LN (*p = 0.1498*).

**Fig. 3:**
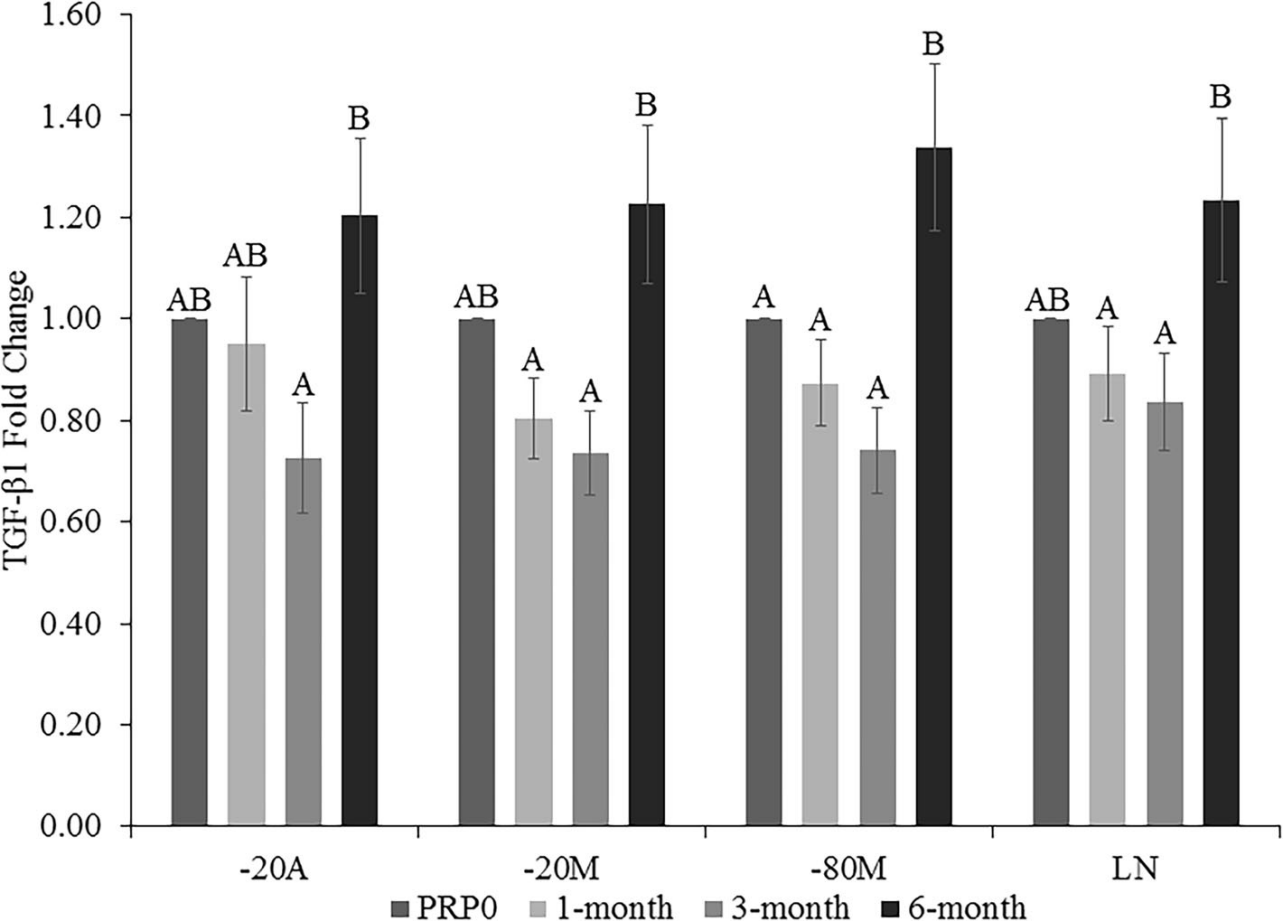
Transforming growth factor-β1 (TGF- β1) fold change in platelet-rich plasma (PRP) at 1, 3, and 6 months of storage in − 20 °C automatic defrost freezer (− 20A), − 20 °C manual defrost freezer (− 20 M), − 80 °C manual defrost freezer (− 80 M), and liquid nitrogen (LN) compared to baseline fresh activated PRP at time 0 (PRP0). All samples were activated with bovine thrombin and calcium chloride prior to growth factor quantification at each time point. Bars represent mean (*n* = 6) ± standard error. Differing letters indicate a significant difference between groups within storage method, using a repeated measures ANOVA with a Tukey post-hoc test, *p* < 0.05

There was no difference in TGF-β1 concentration after 1 and 3 months in any storage condition, compared to PRP0 (Fig. 3). After 6 months of storage in -20A (*p < 0.0085*), there was a significant increase in TGF-β1 compared to 3 months. In -20 M (*p < 0.0014*) and LN (*p < 0.0107*), there was a significant increase following 6 months of storage, compared to 1 and 3 months. When stored in -80 M (*p < 0.0008*), TGF-β1 significantly increased at 6 months, compared to all other time points.

**Fig. 4:**
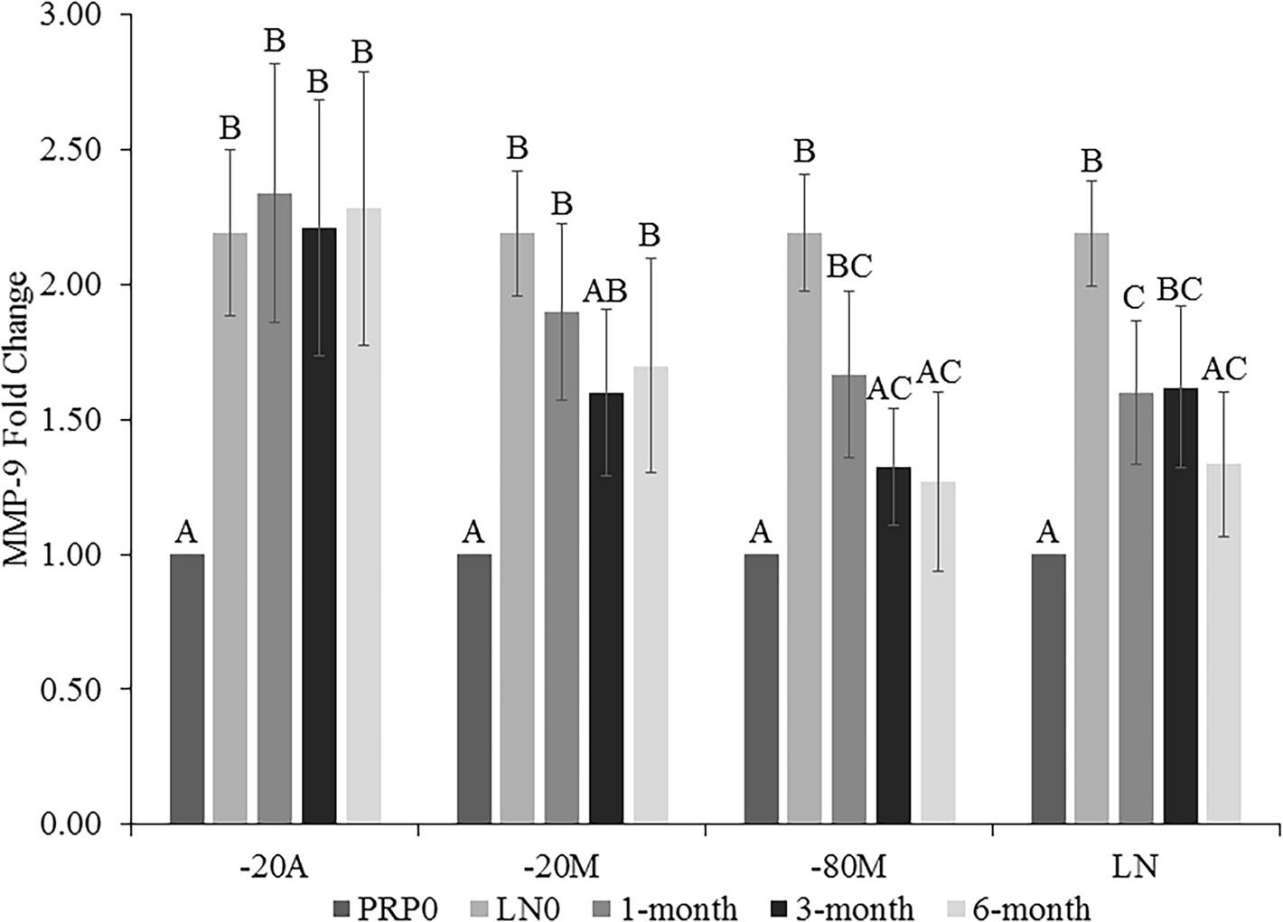
Matrix metalloproteinase-9 (MMP-9) fold change in platelet-rich plasma (PRP) at 1, 3, and 6 months of storage in -20 °C automatic defrost freezer (− 20A), −20 °C manual defrost freezer (−20 M), − 80 °C manual defrost freezer (− 80 M), and liquid nitrogen (LN) compared to baseline fresh activated PRP at time 0 (PRP0) and snap-frozen PRP at time 0 (LN0). All samples were activated with bovine thrombin and calcium chloride prior to growth factor quantification at each time point. Bars represent mean (*n* = 6) ± standard error. Differing letters indicate a significant difference between groups within storage method, using a repeated measures ANOVA with a Tukey post-hoc test, *p* < 0.05

The concentration of MMP-9 did not differ at any time point compared to LN0 in -20A and -20 M, but significantly increased compared to PRP0 (− 20A *p < 0.0013;* − 20 M *p < 0.*0009) with the exception of -20 M at 3 months (Fig. 4). There was no difference in MMP-9 in LN0 and in -80 M after 1 month, which were both significantly increased compared to PRP0 (*p < 0.0003)*. However, after 3 and 6 months of storage in -80 M, MMP-9 significantly decreased compared to LN0 and did not differ from PRP0. Finally, MMP-9 concentrations in LN (*p < 0.0001*) storage were intermediary to PRP0 and LN0 concentrations, with the 1-, 3- and 6-month time points not differing from one another. The 1-month aliquot was significantly different from both PRP0 and LN0, while 3 months was increased compared to PRP0 and 6 months was decreased compared to LN0.

The intra-assay variability was 3.80% for IGF-1, 3.85% for PDGF-BB, 4.69% for TGF-β1, and 4.25% for MMP-9 ELISAs. The inter-assay variability was 4.36% for IGF-1, 1.81% for PDGF-BB, 1.62% for TGF-β1, and 7.74% for MMP-9 ELISAs.

## Discussion

The principle objective of this study was to investigate the effect of freezing on PRP protein concentrations compared to fresh PRP and the optimal type of frozen storage and duration of storage. We approached this by first investigating the effect of a single snap-freeze thaw cycle on concentrations of 3 commonly measured growth factors (PDGF-BB, TGF-β1, IGF-1) and a catabolic enzyme (MMP-9) in PRP. Our results supported our first hypothesis that a single snap freeze-thaw would not affect protein concentrations for PDGF-BB and IGF-1. While TGF-β1 was significantly decreased in LN0 compared to PRP0, this represented a decrease of < 8% which is unlikely to be a clinically relevant change. The 2.2-fold increase in MMP-9 in LN0 compared to PRP0 did not support our first hypothesis. In retrospect, this result should have been anticipated since MMP-9 is principally found in WBC, and has been shown to correlate with neutrophil concentration in human blood [7]. Therefore, MMP-9 would be released upon lysis of WBC in frozen-thawed PRP, while much of this enzyme would remain sequestered in WBC in fresh activated PRP.

Our second hypothesis was not supported by the results of this study. We used four different freezing options that would be available to the general practitioner or to referral hospitals. We expected that the frequent cyclical temperature changes in a standard household automatic defrost freezer would lead to greater protein loss due to denaturation, and that protein concentrations would remain stable as long as PRP was stored frozen at a consistent temperature. The results of this study showed that the effect of temperature and time in storage differs for each protein. This is the first study to investigate the effect of long-term frozen storage of PRP on protein concentrations without additional manipulation of the PRP. There are established methods for long term stabilization of platelets and the proteins present in PRP using dimethyl sulfoxide (DMSO), or sugars such as trehalose and subsequent freeze-drying [20, 21]. These methods were developed for the purpose of platelet transfusion since the shelf-life of platelet concentrates for transfusion is mere days [20]. These techniques are effective and equine freeze-dried platelets have been produced and shown to be biologically comparable to PRP in vitro [10]. However, the additional platelet stabilization steps for frozen storage are cumbersome, require additional processing and special equipment, and are not practical on a small scale [21].

Scant data on the effect of simple freezing on protein concentrations in PRP following short-term storage are available in the scientific literature. Shiga et al. compared concentrations of growth factors from human PRP, including TGF-β1 and PDGF, at the time of preparation and at several time points over 8 weeks, when stored at room temperature, − 80 °C, and freeze-dried [17]. Similar to our study, all specimens were activated with CaCl_2_ and thrombin prior to protein quantitation. The authors reported results that were similar to ours in that PDGF was no different after 4 weeks of storage in -80 °C, but concentrations decreased thereafter. In contrast, their TGF-β1 results differ from ours as concentrations of TGF-β1 decreased at 8 weeks in human PRP, while there was no decrease in TGF-β1 in equine PRP stored at − 80 °C in the present study. Hosnuter et al. determined concentrations of IGF-1, PDGF, and TGF-β1 in human PRP stored up to 14 days at -20 °C and subsequently activated with CaCl_2_ [22]. A decrease in all 3 growth factors was found in the human PRP in this short-term study. While our first storage time-point was later at 1-month, the results of our study were consistent with the results for PDGF and IGF-1, yet TGF-β1 differed once again.

One of the key similarities between our study and the 2 studies described above is that the fresh PRP and the frozen-thawed PRP were activated prior to analysis. Growth factors in platelets are sequestered in platelet alpha-granules and activation is required for their release. Therefore, without activation, fresh PRP would have very low concentrations of growth factors which would be a misleading baseline for comparison [23]. Previous studies comparing platelet activation using CaCl_2_ and thrombin in fresh activated PRP and frozen activated PRP found that 80% of platelets were activated using both methods based on flow cytometry [17]. Textor et al. previously reported an inability to quantitate growth factors in PRP following activation with a combination of CaCl_2_ and bovine thrombin due to salting out of the growth factors [13]. However, we and others [14] did not encounter this issue.

Aside from the difference in species, the key difference between this study and the 2 short-term studies described above, are that the previous studies only quantitated the growth factor contained in the releasate after clotting. However, it has been shown with a variety of activators that a significant amount of growth factors are sequestered in the fibrin clot that forms following activation of the platelets in PRP [13]. Our goal was to determine the total amount of growth factor potentially available in the PRP, so we combined the supernatant with the homogenate of the fibrin clot for analysis. It is possible that the polymerization of fibrin is altered after a freeze-thaw and this may in turn alter the distribution of growth factors between the fibrin clot and the releasate. Further work would be needed to determine if this could explain the differences between studies.

The differences in change in concentration of the various proteins measured over time in frozen storage were not expected. Perhaps most surprising, was the significant decrease in IGF-1 by > 58% at all times in all conditions compared to PRP0. Accurately determining the effects of freezing on proteins is technically challenging due to the solidification of the aqueous portion of protein suspensions. The 3 principle mechanisms that degrade proteins in frozen storage are cold denaturation, freeze concentration of solutes (resulting in pH, viscosity, and interface changes), and ice-induced partial unfolding of protein structure [24]. The duration of frozen storage, the storage temperature, and the freezing and thawing rate are all important factors that affect stability of frozen proteins [24]. Unfortunately, the majority of research in this field is limited to the study of proteins in aqueous solutions. One potential explanation for the dramatic decline in IGF-1 with frozen storage compared to the other proteins measured in this study, is that IGF-1 is produced in the liver and is predominantly free in the plasma, while all other proteins evaluated in this study are sequestered within alpha-granules in platelets (PDGF and TGF-β) or within WBC (MMP-9). The cell membrane may protect these proteins from some of the freeze induced changes that degrade IGF-1. Further study of intracellular proteins under freezing conditions would be needed and is beyond the scope of this study. The increase in TGF-β1 after 6 months of storage was also an unexpected result. We verified this result by repeating analysis with new aliquots and reagents (Additional file 1). It is possible that this result could be due to variations in thrombin activity despite using the same concentration for all samples. Also, one horse had more extreme results that the other horses in the study but did not differ from the mean by greater than 2 standard deviations, and could not justifiably be removed from data analysis. Finally, the TGF-β1 ELISA only measures active protein and an acid activation step is performed prior to analysis. It is possible that a conformation change occurred after 6 months of storage that increased the amount of active TGF-β1 in the sample, although this is less likely.

The principle limitation of this study is that a small subset of the massive number of bioactive factors present in PRP were quantified [5]. Given the protein specific responses to frozen storage of PRP, it is likely that a broader scope of analysis would reveal even more complexity. However, we were limited to the proteins with ELISAs known to be validated for use in the horse. Another limitation is that we only investigated the effect of freezing and frozen storage on total protein concentrations. Further research will be needed to determine the biologic effects of using frozen-thawed PRP compared to fresh PRP in vivo in tissues of interest.

## Conclusions

In conclusion, a single snap freeze-thaw will result in an immediate increase in the amount of catabolic MMP-9 in PRP containing WBC without a clinically relevant change in the concentrations of PDGF, TGF-β1, or IGF-1. Further, PRP is best stored at -80 °C for 1 month, or in LN for up to 6 months for preservation of PDGF and TGF-β1 concentrations. However, if IGF-1 is thought to be an essential growth factor for the intended therapeutic application of PRP, then fresh PRP should be used in these cases. Finally, for research applications, IGF-1 should be measured immediately, while PDGF-BB and TGF-β1 may be quantitated after up to 6 months of storage in liquid nitrogen. The act of freezing itself increases the concentration of MMP-9 and this must be taken into consideration if frozen samples are to be used to quantitate MMP-9 concentrations, which will not accurately reflect the concentrations of MMP-9 applied to tissues or cells if fresh PRP is used for culture or in vivo studies.

## Methods

### Study population

Six healthy adult Thoroughbred horses of mixed sex, between 6 and 8 years of age, were used for this study. Normal health status was confirmed by physical examination and complete blood count (CBC). Horses did not receive any medications or vaccinations for at least 2 weeks prior to starting the study. Animal use was approved by the University of Florida Institutional Animal Care and Use Committee (IACUC).

### PRP preparation and storage

Horses were sedated with 200 mg of xylazine hydrochloride (Rompun®) in the right jugular vein. The left jugular vein was clipped and aseptically prepared. Venous blood was aseptically collected into 13 mL of acid-citrate-dextrose (ACD-A) anti-coagulant to a total volume of 130 mL using an 18-gauge, 1.5-in. needle. Preparation of PRP was carried out using the SmartPReP 2 system (Harvest Technologies) as previously described [10]. Briefly, 60 mL of anti-coagulated whole blood was transferred to the disposable container, centrifuged, and resuspended according to manufacturer instructions to yield 10 mL of PRP. Two kits were prepared for each horse generating a total of 20 mL of PRP per horse. Volumes of PRP were combined and mixed by inversion prior to aliquoting to ensure consistent aliquots within each horse. Aliquots of PRP and anti-coagulated whole blood were submitted for automated hematology using the Siemens Advia hematology analyzer, which has been previously validated for equine PRP [25]. To prevent platelet agglutination which can falsely lower platelet counts, prostaglandin E_1_ was added to the aliquots submitted for hematology, to achieve a concentration of 10 μg of PGE_1_/mL as previously described [12]. The remaining PRP was aliquoted for the following groups: PRP0, LN0, and 1-, 3-, and 6-month aliquots for each storage condition (− 20A, − 20 M, − 80 M, and LN).

Aliquoted samples of PRP from each horse were stored in − 20A, −20 M, − 80 M, and LN until needed for protein quantification at 1, 3, and 6 months from the time of initial PRP preparation. All samples were placed in their respective storage conditions within 2 h of PRP preparation. Previous studies with equine and human blood have shown that concentrations of TGF-β1, PDGF-BB, and MMP-9 do not change within 4–6 h from the time of PRP preparation [26, 27]. The -20A was monitored using an Elitech RC-5 USB temperature data logger, to record temperature fluxes. The data logger was set to record temperature every 15 min and temperature data was downloaded, evaluated and saved throughout the study. All other freezers were monitored continuously on the institutional alarm system.

### PRP thawing and activation

The LN0 sample was activated after being snap-frozen in liquid nitrogen for 5 min and thawed for 5 min in beads warmed to 37 °C. The PRP0 sample was activated at the same time as its paired LN0 sample. For stored PRP samples, aliquots were removed from each storage condition at the designated time point, allowed 5 min to thaw in beads warmed to 37 °C, and then activated. All PRP activation was performed using 1000 IU of lyophilized bovine thrombin resuspended in 1 mL of 10% calcium chloride (CaCl_2_). The CaCl_2_-bovine thrombin solution was added at a 1:10 ratio of activator to PRP, and the samples gently pipetted to ensure thorough mixing. Samples were incubated at 37 °C for 30 min and then immediately processed for protein quantitation.

### Protein quantification

Samples were centrifuged at 1000 x *g* for 10 min after incubation and the clot releasate collected. A tissue homogenizer was then used to liquefy the remaining clot in order to collect all potential measureable proteins sequestered in the clotted PRP. The homogenate was then added to the clot releasate and used immediately for protein quantification via ELISA. Platelet derived growth factor-BB (Human PDGF-BB Quantikine® ELISA), TGF-β1 (Human TGF-β1 Quantikine® ELISA), IGF-1 (Human IGF-1 Quantikine® ELISA), and MMP-9 (Equine MMP-9 RayBio® ELISA) concentrations were determined in duplicate according to manufacturer’s instructions. The PDGF-BB, TGF-β1, and IGF-1 ELISA kits have been validated and used in the horse previously [12, 15, 28]. The MMP-9 kit is specific for equine MMP-9 and validated by the manufacturer for equine plasma samples. Samples were acid activated for the TGF- β1 assay. Standard curves were created using the standards supplied in each ELISA kit. All plates were analyzed using a Fluostar Optima ELISA plate reader, with wavelength absorption set at 450 nm, with correction wavelength set at 540 nm for IGF-1, PDGF-BB, and TGF-β1.

### Data analysis

Data collected from CBCs included platelet and WBC concentrations and hematocrit in whole blood and PRP. The fold-change was calculated for all frozen-thawed PRP samples compared to PRP0. Statistix 10 software was used for statistical analysis. Descriptive statistics were generated, and normality determined using the Shapiro-Wilk test. Paired t-tests were used to compare hematology parameters between whole blood and PRP, and fold-change in protein concentrations between PRP0 and LN0. A repeated measures ANOVA and Tukey post-hoc test for multiple comparisons was used to compare the fold-change in protein concentrations over time within a storage method. Significance was set at *p < 0.05*.

## Additional file

Additional file 1: Comparison of original and repeated 6 month transforming growth factor-β1 (TGF-β1) concentrations for each frozen storage condition. Due to the unexpected increase in TGF-β1 concentration at 6 months of frozen storage, the analysis was repeated with new aliquots, new reagents, and a new ELISA kit. The results were compared to the initial analysis presented in the results of the manuscript and were found to be no different. (PDF 182 kb)

## Data Availability

The datasets used and/or analyzed during the current study are available from the corresponding author on reasonable request.

## Abbreviations

-20 M: -20 °C manual defrost freezer
-20A: -20 °C automatic defrost freezer
-80 M: -80 °C manual defrost freezer
IGF-1: Insulin-like growth factor-1
LN: Liquid nitrogen
LN0: Snap frozen-thawed and activated PRP
MMP-9: Matrix metalloproteinase-9
PDGF-BB: Platelet-derived growth factor-BB
PRP: Platelet-rich plasma
PRP0: Fresh-activated PRP
RBC: Red blood cell
TGF- β1: Transforming growth factor-β1
WBC: White blood cell
