# Supplementary material for: The effect of four different freezing conditions and time in frozen storage on the concentration of commonly measured growth factors and enzymes in equine platelet-rich plasma over six months
Source: BMC Vet Res. 2019 Aug 14;15:292. doi: 10.1186/s12917-019-2040-4 (PMC6694589; doi:10.1186/s12917-019-2040-4)
Supplement: Supplementary file 1 — Comparison of original and repeated 6 month transforming growth factor-β1 (TGF-β1) concentrations for each frozen storage condition. Due to the unexpected increase in TGF-β1 concentration at 6 months of frozen storage, the analysis was repeated with new aliquots, new reagents, and a new ELISA kit. The results were compared to the initial analysis presented in the results of the manuscript and were found to be no different. (PDF 182 kb) [file 12917_2019_2040_MOESM1_ESM.pdf]

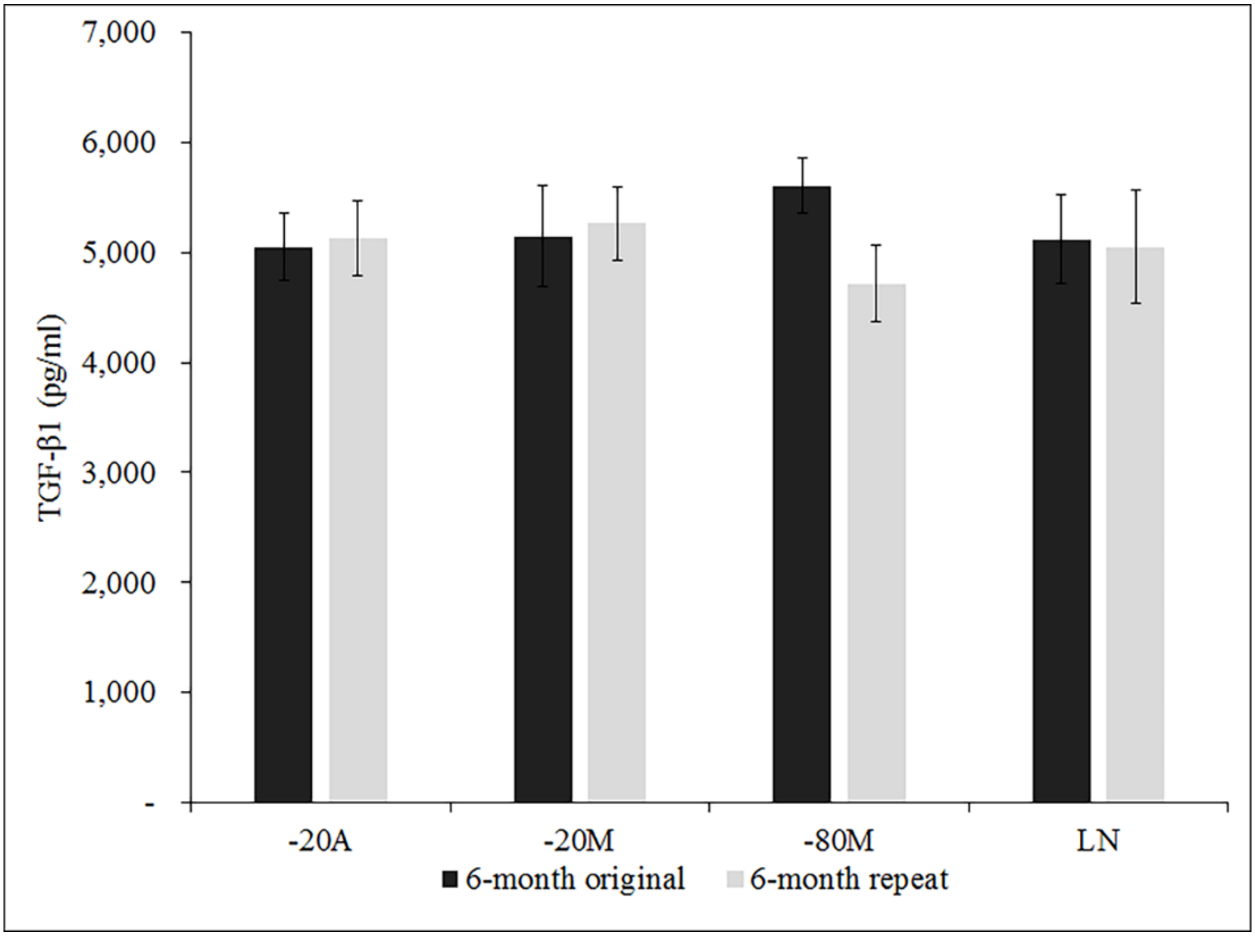

Additional file 1: Transforming growth factor-β1 (TGF- β1) concentrations at original 6-month and repeat 6-months of storage in -20°C automatic defrost freezer (-20A), -20°C manual defrost freezer (-20M), -80°C manual defrost freezer (-80M), and liquid nitrogen (LN). All samples were activated with bovine thrombin and calcium chloride prior to protein quantification. Bars represent mean (n=6) ± standard error. There was no significant difference between original and repeat data when compared using a paired t-test, $p<0.05$.
